# The protective role of vitamin C against linezolid-induced hepato-renal toxicity in a rat model

**DOI:** 10.3389/fphar.2025.1551062

**Published:** 2025-06-02

**Authors:** Shaimaa M. Azzam, Hend Mohamed Anwar, Ahmed H. Abd El-Slam, Marwa S. M. Diab, Halima Mohamed Ibrahim, Abdalrahman Mohammed Yousef, Fatma Mahmoud Sabry, Ibrahim A. Darwish, Kalidasan Kaliyamoorthy, Gad Elsayed Mohamed Salem, Heba M. A. Elsanhory

**Affiliations:** ^1^ Department of Biochemistry, Egyptian Drug Authority (EDA), Formerly National Organization for Drug Control and Research (NODCAR), Giza, Egypt; ^2^ Department of Forensic Medicine and Clinical Toxicology, Faculty of Medicine, Al-Azhar University, Cairo, Egypt; ^3^ Cell Biology and Histology, Molecular Drug Evaluation Department, Egyptian Drug Authority (EDA) Formerly National Organization for Drug Control and Research (NODCAR), Giza, Egypt; ^4^ Department of Physiology, Egyptian Drug Authority (EDA), Formerly National Organization for Drug Control and Research (NODCAR), Giza, Egypt; ^5^ Department of Pharmacology, Egyptian Drug Authority (EDA), Formerly National Organization for Drug Control and Research (NODCAR), Giza, Egypt; ^6^ Department of Pharmaceutical Chemistry, College of Pharmacy, King Saud University, Riyadh, Saudi Arabia; ^7^ Atal Centre for Ocean Science and Technology for Islands, National Institute of Ocean Technology, Port Blair, India; ^8^ Department of Microbiology, Egyptian Drug Authority (EDA), Formerly National Organization for Drug Control and Research (NODCAR), Giza, Egypt; ^9^ Pharmacology and Toxicology Department, Faculty of Pharmacy, Sinai University, East Kantara Branch, El Ismailia, Egypt

**Keywords:** linezolid, vitamin C, hepatotoxicity, nephrotoxicity, oxidative stress, inflammation

## Abstract

This study investigated the protective effects of vitamin C against linezolid-induced hepatotoxicity and nephrotoxicity in rats using biochemical, histopathological, and immunohistochemical methods. Twenty-four rats were divided into four groups: control, linezolid (100 mg/kg/day), vitamin C (100 mg/kg/day), and a combination of vitamin C and linezolid for 14 days. Linezolid treatment resulted in lactic acidosis, increased serum levels of AST, ALT, urea, creatinine, and albumin, and oxidative stress characterized by decreased catalase activity and reduced glutathione (GSH) associated with increased nitric oxide (NO) malondialdehyde (MDA). Pro-inflammatory markers (IL-1β, TNF-α) and renal CD68 expression also increased. Linezolid disrupted autophagy (reduced Beclin-1 levels) and induced apoptosis through hyperactivation of Wingless/integrated (Wnt) signaling (increased Wnt 7a and Wnt 10a expression). Treatment with vitamin C alleviated linezolid side effects by reducing AST, ALT, urea, creatinine, lactic acid, IL-1β, TNF-α, NO, MDA, and Wnt signaling markers while increasing albumin, catalase, GSH, and Beclin-1 levels. Histopathological and immunohistochemical analyses confirmed significant protection of liver and kidney tissues in rats co-administered vitamin C with linezolid. These results suggest that vitamin C can effectively alleviate hepatotoxicity and nephrotoxicity caused by linezolid.

## 1 Introduction

Linezolid is an oxazolidinone antibiotic that acts against Gram-positive bacteria by disrupting their protein production (Abd El Latif et al., 2019). LZD was incorporated into treatment protocols for pneumonia linked to COVID-19, as recommended by the National Institute for Health and Care Excellence in the UK in May 2020 (Brenciani et al., 2022; Duployez et al., 2020; Li Q. et al., 2020). Although linezolid is usually well tolerated for brief periods of time, negative side effects can still happen with short-term exposure. Linezolid’s efficacy in treating multiple drug-resistant tuberculosis (MDR-TB) was underlined by Oehadian et al. (2022), who also pointed out that it is linked to hematologic damage and mitochondrial dysfunction and suggested routine monitoring. A case study presented a 79-year-old woman who experienced acute liver failure following a single day of linezolid medication; this condition improved following cessation and supportive care (Chokshi et al., 2023). Another study reported that a 65-year-old woman with diabetes experienced hepatic encephalopathy following a week of linezolid treatment; this condition improved once the medication was stopped (Upadhyay et al., 2024). According to Ramesh et al. (2024), a patient who experienced vomiting, dyspnea, hypotension, and high anion gap metabolic acidosis—all of which were likely caused by linezolid-induced lactic acidosis—improved after stopping the medication.

Clinical studies have reported that linezolid therapy may potentially provoke hepatic impairment (De Bus et al., 2010; Ikuta et al., 2011). LZD resulted in liver damage through elevating concentrations of transaminases such as alanine aminotransferase (ALT) and aspartate aminotransferase (AST), alongside increasing alkaline phosphatase (ALP), lactate dehydrogenase (LDH), serum bilirubin, and lactate levels in rats (Vivekanandan et al., 2018). Additionally, LZD caused renal insufficiency and increased risk of thrombocytopenia (Matsumoto et al., 2009), as well as increasing serum urea levels (Abd El Latif et al., 2019). LZD induced lactic acidosis by inhibiting mitochondrial protein synthesis (Su et al., 2011). LZD provoked oxidative stress, which plays a crucial role in initiating apoptosis. Increasing evidence supports the notion that oxidative stress and apoptosis are closely linked to both physiological processes and the pathogenesis of various chronic diseases (Kannan and Jain, 2000). Some studies have suggested that activation of the Wnt/β-catenin signaling pathway can induce apoptotic cell death, at least in part, through a caspase-dependent mechanism (Deng et al., 2014).

Vitamin C, a potent antioxidant, can alleviate various organ injuries by reducing oxidative stress. This antioxidant can also mitigate drug-induced apoptosis by neutralizing superoxide anions generated by dysfunctional mitochondria (Chiu et al., 2016) and reduce DNA damage by lowering TNF levels and suppressing the activation of the caspase cascade (Ramanathan et al., 2005). It has been reported that vitamin C exhibits hepatoprotective effects by increasing serum levels of SOD and GSSG, indicating a reduction in oxidative stress and decreased serum levels of liver enzymes (Abdulrazzaq et al., 2019). Furthermore, vitamin C has been shown to mitigate vancomycin-related renal injury by reducing oxidative stress, cellular apoptosis, and inflammation (He et al., 2021).

This study aimed to investigate the protective role of vitamin C in mitigating hepatotoxicity and nephrotoxicity induced by linezolid in an experimental rat model. Through a comprehensive approach encompassing biochemical, histopathological, and immunohistochemical analyses, the research sought to elucidate the mechanisms underlying linezolid-induced oxidative stress, inflammation, and apoptosis. By assessing the interplay between oxidative stress markers, inflammatory mediators, and apoptotic pathways, this study aimed to provide valuable insights into the therapeutic potential of vitamin C as an antioxidant in reducing drug-induced organ toxicity.

## 2 Materials and methods

### 2.1 Animals

This study was conducted on male Wistar albino rats provided by the National Organization for Drug Control and Research (NODCAR).

### 2.2 Drugs

Tablets of linezolid were acquired from Global Napi Pharmaceuticals Company in Egypt. Vitamin C capsules were purchased from Hikma Pharma S.A.E Company, Egypt.

### 2.3 Experimental design

The present experimental study was carried out on Wistar albino rats aged 3–4 months and weighing between 170 g and 200 g. The animals were obtained from a pure strain to maintain a consistent and stable genetic background. The animals were provided with unrestricted access to food and water *ad libitum*. They were housed under controlled conditions of 21°C–24°C, 40%–60% relative humidity, and a 12-h light–dark cycle. All experimental procedures adhered to the guidelines of the institutional Ethics Committee and followed recommendations for the ethical care and use of laboratory animals. Efforts were made to minimize any unnecessary disturbances, and the animals were handled with care, avoiding squeezing, pressure, or rough handling. All animal experimentation protocols were reviewed and approved by the Ethics Committee of the National Organization for Drug Control and Research (NODCAR), following the organization’s established guidelines. The approval reference number is NODCAR/II/40/2022.

Experimental animals were randomly assigned into four groups of six rats in each group:

1. Control group: Normal saline was administered orally to the animals.
2. Linz group. Linezolid (dissolved in normal saline, 100 mg/kg/day) was given to the animals. It was administered for 14 days via oral gavage (Abd El Latif et al., 2019).
3. Vit. C group: 100 mg/kg of vitamin C dissolved in normal saline was given to the animals each day. It was administered for 14 days via oral gavage (Ergul et al., 2010).
4. Linz+ Vit.C group: The animals were given vitamin C dissolved in normal saline at a dose of 100 mg/kg/day prior to administrating the dose of linezolid (dissolved in normal saline, 100mg/kg/day) via oral gavage for consecutive 14 days.

**FIGURE 1:**
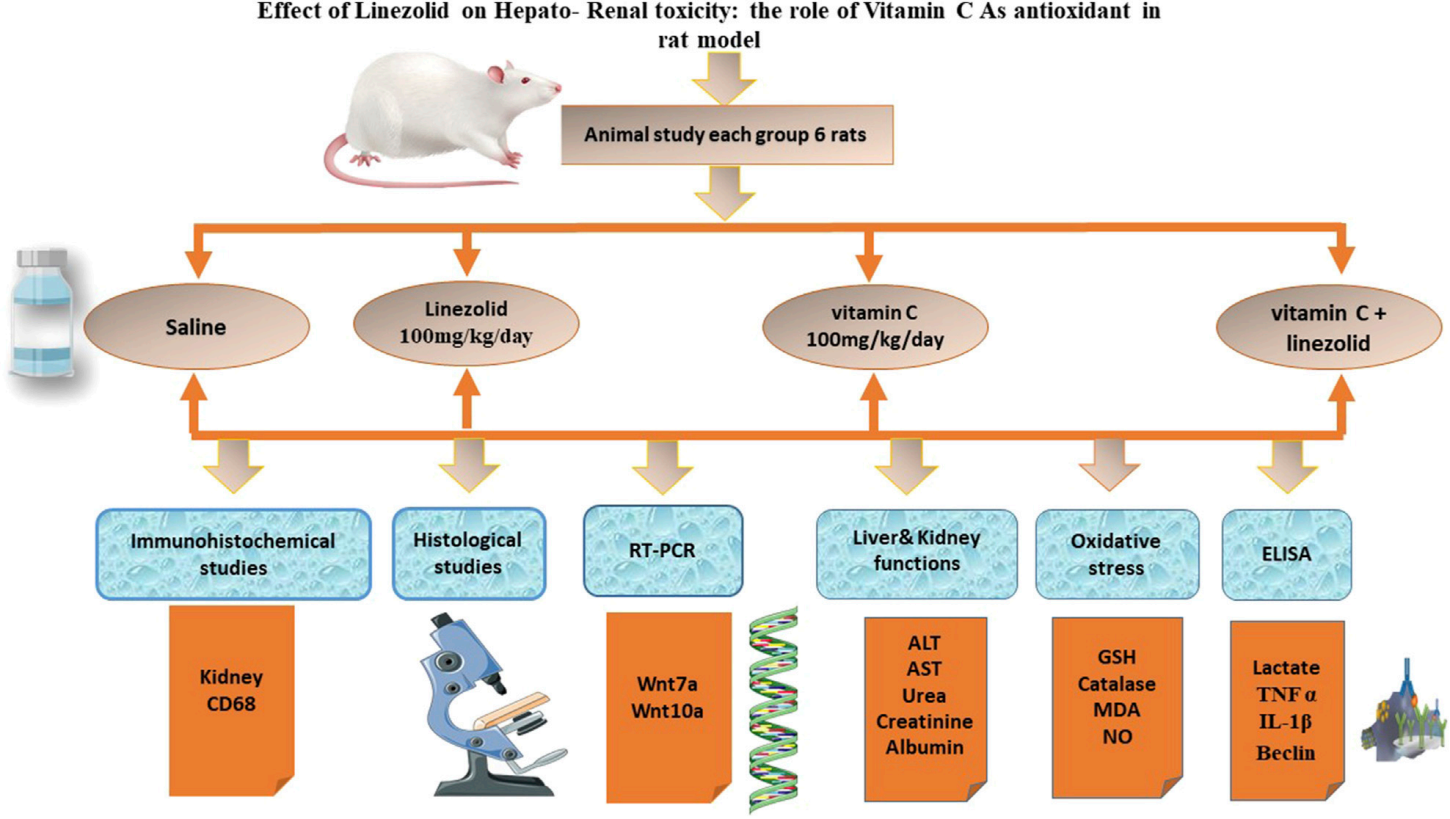
Experimental design for evaluating the protective effects of vitamin C against linezolid-induced toxicity.

The experimental design to evaluate the protective effects of Vitamin C against linezolid-induced toxicity is illustrated in Figure 1.

After the final procedure, the rats were anesthetized to facilitate sample collection 24 h later. The anesthesia was administered intraperitoneally, using ketamine at a dose of 90 mg/kg and xylazine at 10 mg/kg. Both solutions were carefully pre-warmed to 37°C to ensure proper administration and minimize stress to the animals. Blood samples were taken for serum separation after anesthesia was administered. Immediately after, the liver and kidney were removed and kept at −80°C for further biochemical examination. ELISA testing involved homogenizing a fraction of the tissues in a lysis solution supplemented with protease and phosphatase inhibitors (200 mM NaCl, 5 mM EDTA, 10 mM Tris, 10% glycerol; pH 7.4). For additional examination, the supernatant was kept at −80°C after the homogenate was centrifuged at 10,000 g for 15 min at 4°C. Using 10% formalin-buffered saline, tissues from each group were kept for histological and immunohistochemical analysis. Finally, the euthanized animals were frozen until they could be cremated.

### 2.4 Biochemical analysis

#### 2.4.1 Estimation of liver and kidney functions

Serum levels of aspartate aminotransferase (AST) and alanine aminotransferase (ALT) were determined using reagent kits provided by Bio Diagnostics (Catalog numbers AL 1031 and AS 1061). Biochemical kits from Spin React, Barcelona, Spain, were utilized to analyze aspartate concentrations (Salem et al., 2023). Colorimetric assay kits from Biomed Diagnostics (Cairo, Egypt) were employed to measure serum concentrations of creatinine, urea, and blood albumin. The specific kits used were the QuantiChrom™ Creatinine Assay Kit (DICT-500), the QuantiChrom™ Urea Assay Kit (DIUR-500), and the QuantiChrom™ BCG Albumin Assay Kit (DIAG-250), respectively. Following the manufacturer’s protocols, the assays were performed using a UV Spector 210 Plus spectrophotometer (Serial No. 1223F1013F) (Khames et al., 2019).

#### 2.4.2 Oxidative stress and antioxidant activity

Liver tissues were isolated by washing, drying, and crushing into tiny pieces in ice-cold isotonic saline and then standardizing in ice-cold. MDA, a sign of lipid peroxidation, was then determined using 1.15% KCl (Uchiyama and Mihara, 1978). Nitric oxide (NO) and GSH levels were determined spectrophotometrically using the method reported by Montgomery and Dymock (1961) Montgomery (1971), with reagent kits purchased from Biodiagnostic Company (Egypt). Serum catalase activity (CAT) was assessed using a spectrophotometric method as outlined by Aebi (1984). A further portion of liver tissue was homogenized in ice-cold sulfosalicylic acid to make a 10% (w/v) homogenate for GSH estimation using the Biodiagnostics kit (Cat no. GR 25 11) using a UV Specord 210 plus Serial. NO. 1223F1013F.

#### 2.4.3 Measurement of serum biomarkers

The serum lactate level was measured using a rat lactic acid ELISA kit (My BioSource Cat No: **MBS720249_48T**). Based on instructions from the manufacturer, the assays were performed by ELIZA HIDEX Serial NO. 321-0311. A colorimetric ELISA kit detected the presence of TNF-IL-1-Beclin. The kits were purchased from MyBiosource (Cat No: MBS7202493), (Catalog No: MBS764668), and (Cat No: MBS2706719), respectively. The assays were conducted by ELIZA HIDEX Serial. NO. 321-0311, according to the manufacturer’s instructions.

### 2.5 Gene expression using RT-PCR

Approximately 30 mg of tissue was preserved in RNA lysis solution at −80°C until genetic analysis. Real-time quantitative reverse transcription PCR (RT-PCR) was employed to assess the expression levels of Wnt7a and Wnt10a genes. Total RNA was extracted from frozen tissue samples using TRIzol reagent, followed by conversion to complementary DNA (cDNA) using SMART Scribe Reverse Transcriptase (Clontech Laboratories, Inc., a Takara Bio Company). RT-PCR reactions were conducted using a Real-Time PCR system (DTlite, DNA Technology, LLC, Moscow, Russia, 117587) and SYBR Green PCR Master Mix in a 25 µL reaction volume. The thermal cycling conditions involved an initial denaturation step at 95°C for 15 s, followed by 40 cycles of denaturation at 95°C for 15 s, annealing at 60°C for 15 s, and extension at 72°C for 45 s. The specific primer sequences for each gene are listed in Table 1 **.** Data analysis was performed using the ABI Prism sequence detection system software and the PE Biosystems Sequence Detection software. The relative expression levels of the target genes were calculated using the comparative Ct method, with GAPDH serving as the reference gene for normalization.

**TABLE 1:** RT-PCR primer sequences.

Gene	Sequence	Accession number
Wnt10a	Forward: 5′CCGACCTGGTCTACTTTGAGA-3′ Reverse, 5′-TCTTGAGACCCAGAGGAGCTT-3′	XM_033112348.1
Wnt7a	Forward: 5′GCGTCTCGCACACTTGCAC 3′ Reverse: 5′CTCCTCCAGGATCTTTCGGC 3′	XM_003826235.4
GAPDH	Forward: 5′GACAGTCAGCCGCATCTTCT3′ Reverse: 5′GCGCCCAATACGACCAAATC3′	XM_003819132.3

### 2.6 Histopathological studies

Male rats were euthanized on the fifteenth experimental day. Small, fresh tissue samples from the liver and kidneys were collected and immediately preserved in 10% formalin solution for at least 24 h. The tissues were then processed using standard paraffin embedding techniques, involving dehydration with increasing ethanol concentrations, clearing with xylene, and embedding in paraffin wax at 60°C. Paraffin blocks were sectioned into 5-μm-thick slices and stained with hematoxylin and eosin (H&E) (Suvarna et al., 2018). To evaluate hepato-renal injury histologically, all tissue sections were examined under an Olympus BH-2 microscope at a 20 µm or 50 µm scale. To assess the severity of liver injury, the presence and extent of congestion, lymphatic infiltration, necrosis, hydropic degeneration, and pyknosis were evaluated. Kidney injury was assessed based on the presence and severity of lymphatic infiltration, tubular degeneration, glomerular fragmentation, and vacuolation. An ordinal scoring method was used to grade the histological changes in liver and kidney tissues among the different groups, according to Meyerholz and Beck (2019). The scale ranges from absent or normal (−) to mild (+) or moderate (++).

### 2.7 Immunohistochemical studies

Kidney tissue sections, 5 μm thick, were mounted on positively charged slides for immunohistochemical staining. The anti-inflammatory marker CD68 was detected using a primary anti-CD68 antibody (Lab Vision, Neomarkers, USA) for 90 min. Subsequently, the immunoperoxidase technique (Vectastain ABC kit; Vector Laboratories, Burlingame, CA) was employed to visualize the antibody-antigen complexes.

### 2.8 Immunohistochemicalimage analysis for CD68

Quantitative immunohistochemical analyses of CD68 expressions in all the examined renal tissues were scored using the Leica Qwin 500 Image Analyzer (Leica Imaging Systems Ltd., Cambridge, London) in the Pathology Department of Cairo University’s Faculty of Veterinary Medicine as outlined by Smolen (1990), where the brown color intensities were expressed as the relative optical density of the DAB reaction. For each animal, CD68-positive immune reactions were quantified in 10 non-overlapping fields per section at a magnification of ×200.

### 2.9 Statistical analysis

Data are presented as mean ± standard deviation (SD). Data were analyzed using IBM SPSS 25. Data were tested for normality using the Shapiro–Wilk test and analyzed using one-way ANOVA followed by *post hoc* test Tukey’s multiple comparisons test to determine statistical differences. Statistical significance for biochemical parameters was set at p ≤ 0.05. GraphPad Prism software (version 6.0) was used for graphical presentation of data.

## 3 Results

### 3.1 Impact of linezolid and/or Vit. C on liver and kidney functions

**FIGURE 2:**
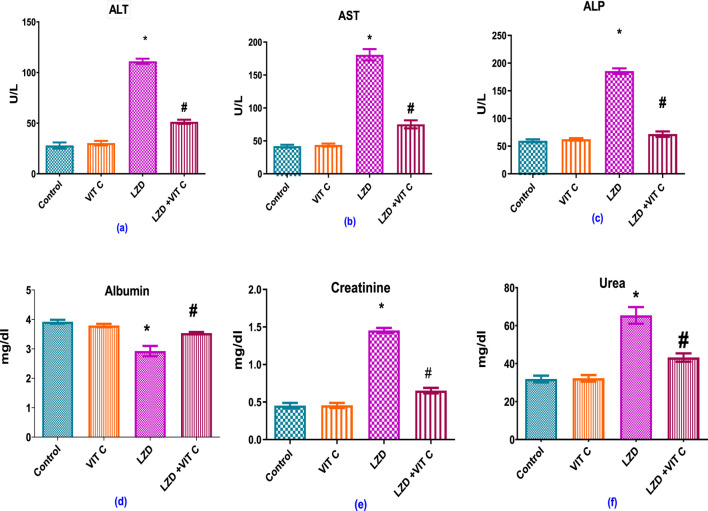
Effects of vitamin C and linezolid on liver and kidney function biomarkers (ALT, AST, ALP, albumin, creatinine, and urea). The data are presented as mean ± SD (n = 6 rats/group). * Significant difference from the control group at p < 0.05; $ Significant difference from the Vit. C group at p < 0.05; # Significant difference from the LZD group at p < 0.05.

The statistical analysis revealed that the administration of linezolid resulted in disruptions to the functioning of the liver and kidneys. Figure 2 shows that, in comparison to the control group liver’s AST, ALT, and ALP values (41.88 ± 2.966 IU/L, 27.92 ± 1.2 IU/L, and 59.58 ± 2.764 IU/L, respectively), the LZD-treated group represented significantly higher levels of hepatic performance parameters (180.8 ± 2.582 IU/L, 111.1 ± 1.05 IU/L, and 185.4 ± 5.125 IU/L, respectively). In addition, rats treated with linezolid had significantly (p ≤ 0.05) lower levels of albumin and higher levels of serum urea and creatinine (2.93 ± 0.175 mg/dL, 1.45 ± 0.034 mg/dL, and 65.38 ± 4.392 mg/dL, respectively) than the control non-treated group (3.92 ± 0.067 mg/dL, 0.45 ± 0.036 mg/dL, and 31.85 ± 1.787 mg/dL, respectively), a finding that suggests compromised kidney function. When vitamin C and linezolid were administered concomitantly, liver and kidney functions parameters were alleviated and measured values approached control levels (75.06 ± 2.074 IU/L, 51.31 ± 0.85 IU/L, and 71.67 ± 4.855 IU/L for AST, ALT, and ALP levels and 3.5 ± 0.039 mg/dL, 0.65 ± 0.034 mg/dL, and 43.19 ± 2.195 mg/dL for albumin, urea, and creatinine levels, respectively) compared to the LZD-treated group (p ≤ 0.05).

### 3.2 Impact of linezolid and/or vitamin C on antioxidant and oxidative stress parameters

**FIGURE 3:**
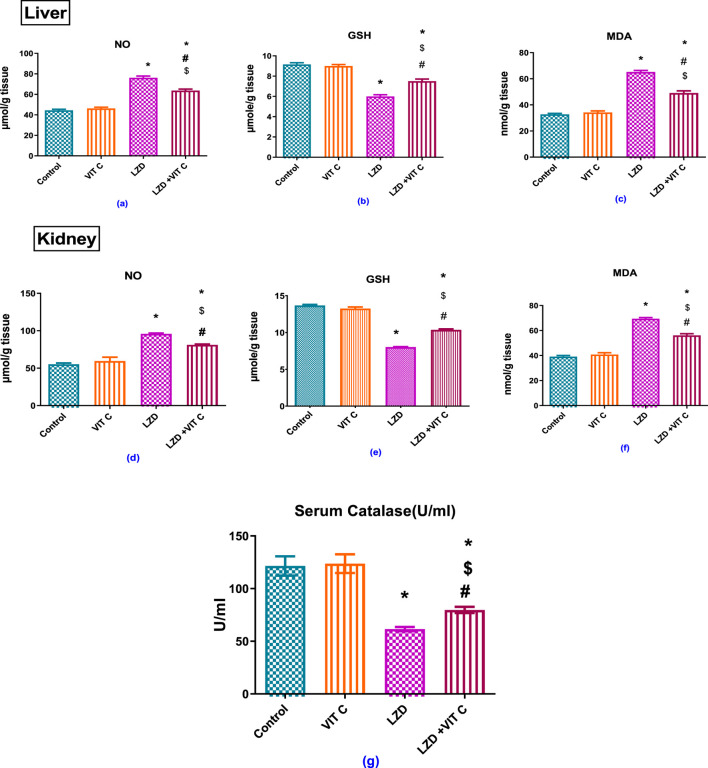
Effects of vitamin C and linezolid on antioxidant and oxidative stress parameters. The data are presented as mean ± SD (n = 6 rats/group). * Significant difference from the control group at p < 0.05; $ Significant difference from the Vit. C group at p < 0.05; # Significant difference from the LZD group at p < 0.05.

As shown in Figure 3, the treatment with linezolid induced a status of oxidative stress represented by significantly (p ≤ 0.05) elevated levels of hepatic and renal NO (76.23 ± 1.677 μmole/g and 95.88 ± 1.065 μmol/g tissue, respectively) versus 44.42 ± 1.054 μmole/g and 55.31 ± 1.56 μmole/g tissue, respectively and lipid peroxidation product, MDA, (65.22 ± 1.116 and 69.51 ± 0.85 nmol/g tissue, respectively) versus 32.76 ± 0.745 nmole/g and 39.15 ± 0.914 nmol/g tissue, respectively. Such effects were associated with reduced hepatic and renal GSH content, which was found to be significantly (p ≤ 0.05) lower than controls (6.003 ± 0.156 μmole/g and 8.03 ± 0.089 μmole/g tissue, respectively) versus 9.17 ± 0.1566 μmole/g and 13.68 ± 0.331 μmole/g tissue, respectively, and depleted serum catalase activity to about one-half (61.60 ± 2.004 U/mL versus 121.5 ± 9.166 U/mL). This state of oxidative stress was ameliorated upon pretreatment with Vit. C as oxidative stress parameters returned to values near to controls.

### 3.3 Impact of linezolid and/or vitamin C on serum lactic acid, Beclin-1, and inflammatory parameters, TNF-α, and IL1β

**FIGURE 4:**
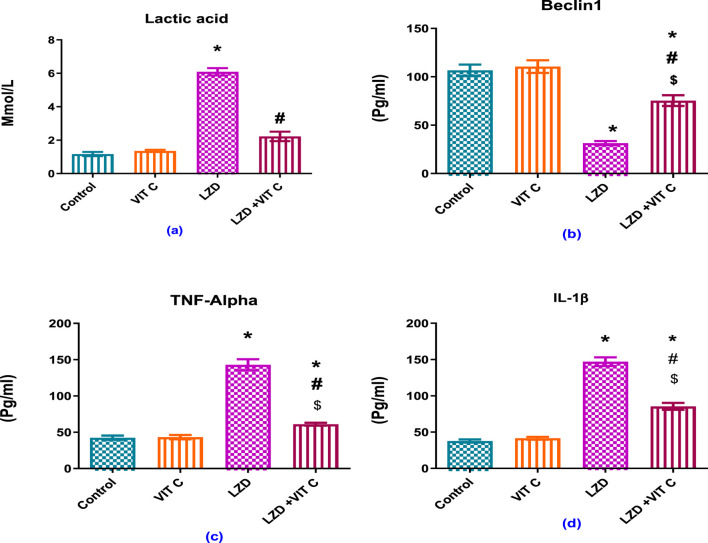
Effects of vitamin C and linezolid on serum lactic acid and inflammation parameters. The data are presented as mean ± SD (n = 6 rats/group). * Significant difference from the control group at p < 0.05; $ Significant difference from the Vit. C group at p < 0.05; # Significant difference from the LZD group at p < 0.05.

As shown in Figure 4, linezolid induced a significant elevation of serum lactic acid level (p ≤ 0.05) to approximately 5-fold (6.090 ± 0.220 mmol/L) compared to the non-treated control group (1.168 ± 0.125 mmol/L), accompanied by significant increase of inflammatory mediators both TNF-α to approximately 3-fold and IL1β to approximately 4-fold (142.9 ± 7.678 pg/mL and 147.0 ± 6.602 pg/mL, respectively) versus controls (42.45 ± 2.928 pg/mL and 37.73 ± 2.193 pg/mL, respectively). The found enhanced inflammation induced by LZD was associated with a significant reduction of Beclin-1 expression (p ≤ 0.05) to about one-third (31.42 ± 2.124 pg/mL) compared to that of the control group (106.7 ± 5.905 pg/mL). Vit. C co-treatment with linezolid provided significant protective effect against inflammation through reduced serum lactic acid (2.227 ± 0.2813 pg/mL), TNF-α (61.05 ± 2.036 pg/mL), and IL1β (85.5 ± 4.826 pg/mL) levels compared to the linezolid-treated group. These levels were slightly above those of the control group (p ≤ 0.05). Vit. C co-treatment with linezolid was also associated with a significant elevation of protective Beclin-1 (75.35 ± 5.525 pg/mL) compared to the linezolid-treated group (p ≤ 0.05).

### 3.4 Impact of linezolid and/or vitamin C on liver and kidney Wnt7a and Wnt10a relative gene expression

**FIGURE 5:**
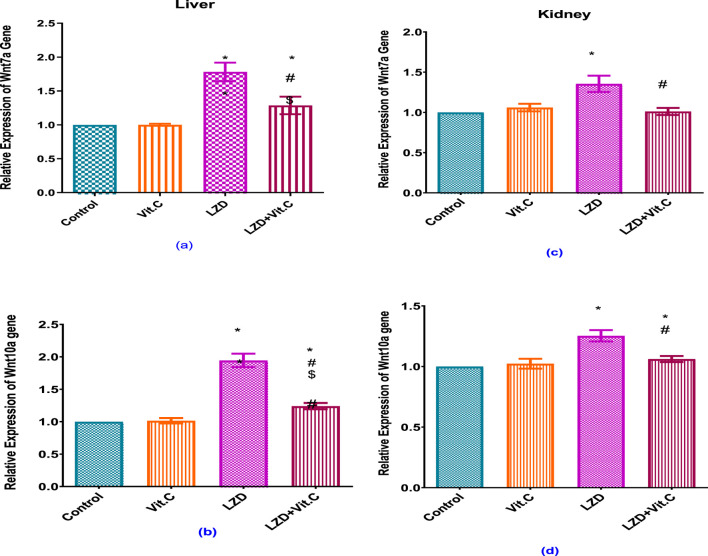
Effects of vitamin C and linezolid on hepatic and renal Wnt7 and Wnt 10 gene expression. The data are presented as mean ± SD (n = 6 rats/group). * Significant difference from the control group at p < 0.05; $ Significant difference from the Vit. C group at p < 0.05; # Significant difference from the LZD group at p < 0.05.

Linezolid treatment caused a significant increase of Wnt7a and Wnt10a relative gene expression in both the liver and kidney of rats compared to rats in the non-treated control group (p ≤ 0.05). Vitamin C co-administration with linezolid resulted in a significant reduction of Wnt7a and Wnt10a relative gene expression in both liver and kidney compared to the linezolid-treated group, but the gene expressions were still significantly higher than those of either the non-treated control or the Vit. C-treated groups (p ≤ 0.05), Figure 5.

### 3.5 Histopathology results of H&E staining method for rat liver

**FIGURE 6:**
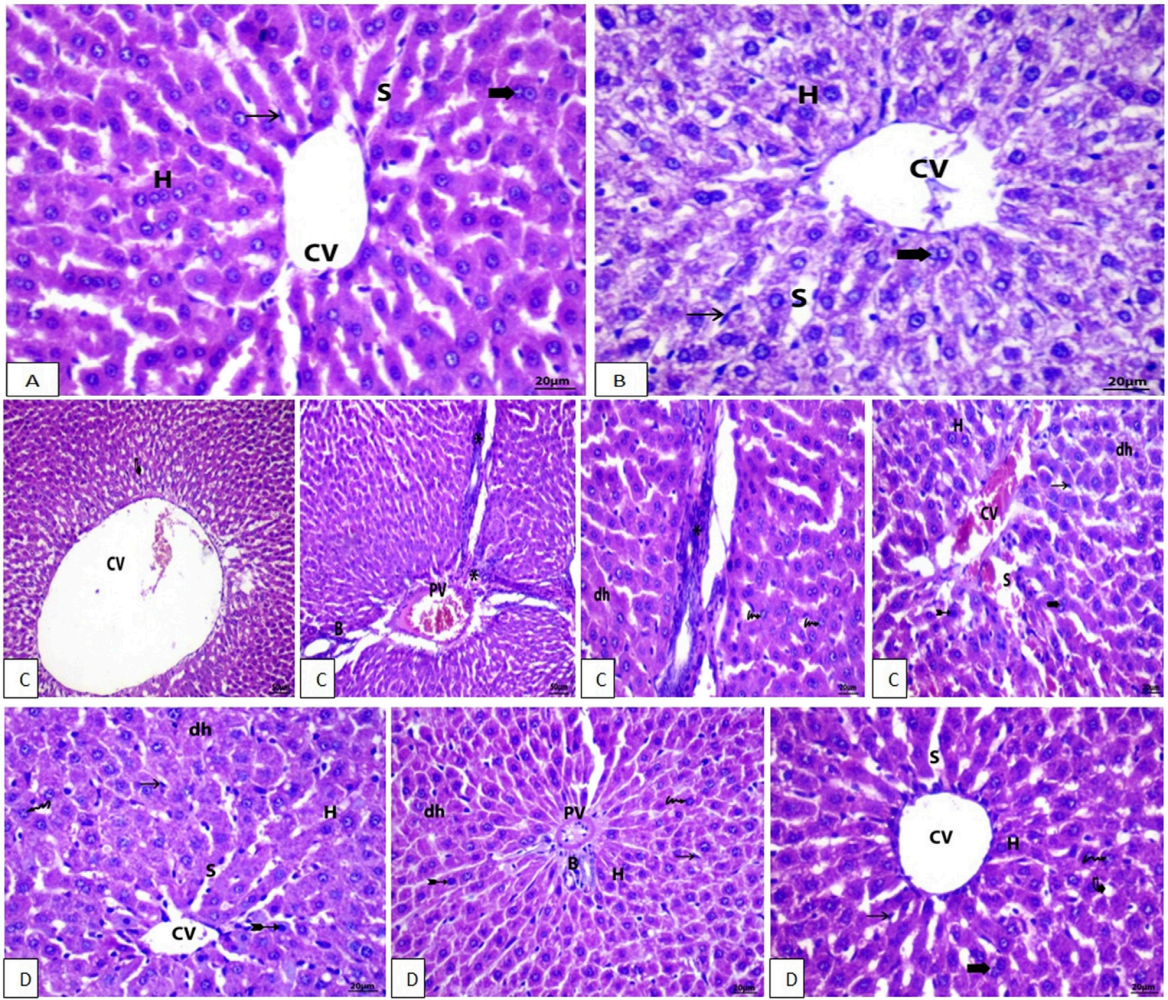
**(A, B)** Photomicrographs of H&E stained liver section of control and Vit. C group, respectively, showing the standard and common structure with the distinctive central vein (CV) and hepatocyte (H) layout (Scale bar = 20). **(C, D)** Photomicrographs of H&E stained liver sections of LZD and (LZD + Vit. C) groups, respectively, **(C)** showing stained liver section with mild fatty degeneration (curved arrow), congestion of the hepatic central vein (CV), and lymphatic infiltration (arrowhead). PV, portal vein; BD, bile duct; CV, central vein; S, sinusoid; asterisk, lymphatic infiltration; Arrow, Kupffer cell; Bifid arrow, pyknotic cell; dh, degenerated hepatocyte; Arrowhead, lymphatic infiltration; Bold arrow, binucleated cell.

A photomicrograph of a liver specimen stained with H&E is shown in Figure 6A. The control rat exhibited a typical central vein and hepatocyte arrangement, as well as a healthy structure with no pathological aberrations. The nuclei of hepatocytes are apparent as dark red structures amid cells, while the cytoplasm appears red. Sinusoids and the normal portal area, as well as hepatic strands extending from the lobule’s margin to the central vein, are normal. There were binucleated cells and Kupffer cells. In the rats treated with Vit. C and linezolid (Group 4) (Figure 6B), hepatic histology is adequate, and the hepatic cords are properly positioned around the sinusoids. Hepatocytes and portal component components appear to be normal. Congestion is at a bare minimum, and no signs of fatty degeneration or mononuclear cell infiltration are seen.

In the linezolid group, liver sections (Figure 6C) show lobular disorganization, nuclear disintegration, fatty degeneration, and disarrayment of regular hepatic cells. In addition, the hepatic central vein was found to be expanded and clogged, and lymphocytic proliferation was noted in the portal area. Examination of liver sections of rats in the linezolid + Vit. C group: Hepatocytes and portal components in Figure 6D show indications of progress where active euchromatic nuclei in hepatocytes appear to be in good condition. There are no inflammatory cell infiltrations around the portal triads in hepatic sections. The congestion is not severe, no fatty degeneration is identified, and only a few degenerated hepatocytes are found. The parameters that indicate the severity of the morphological changes of the liver after different treatments are summarized in Table 2.

**TABLE 2:** Semi-quantitative analysis of histology of liver of rats.

Groups	Congestion	Congested sinusoid	Lymphatic infiltration	Pyknosis	Fatty degeneration (steatosis)
Control	−	−	−	−	−
Vit. C	−	−	−	−	−
Linezolid	++	++	+	++	++
Linezolid + Vit. C	+	+	+	+	+

(−) indicates normal, (+) indicates mild, and (++) indicates moderate.

### 3.6 Histopathology results of H&E staining for renal tissue of rat

**FIGURE 7:**
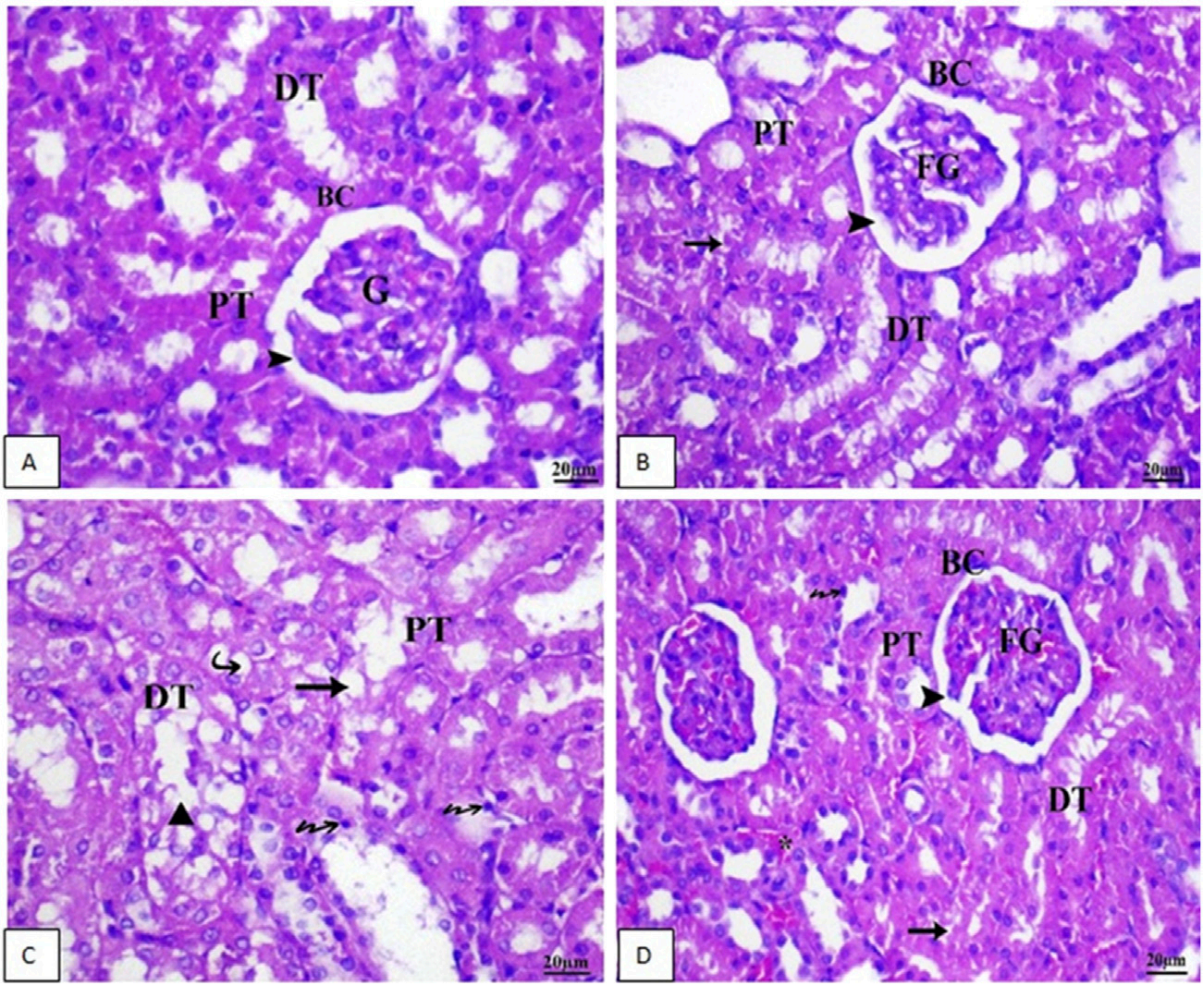
Photomicrographs of renal tissue of rat (scale bar 20 µm) from the control group **(A)** and the Vit. C **(B)** group exhibited normal renal architecture. In contrast, Group 3 **(C)**, the LZD group, displayed rigorous renal injuries distinguished by extensive necrosis, vacuolar deterioration, tubular dilation, epithelial cell shedding, and intraluminal cast development primarily within the proximal tubules. As a final point, **(D)** LZD + Vit. C (Group 4) shows a marked improvement in the histological picture. BC, Bowman’s capsule, G, glomeruli; DG, degenerated glomeruli; EG, enlarged glomeruli; SG, shrunken glomeruli; CG, congested glomeruli; PT, proximal tubule; DT, distal tubule; Arrowhead, capsular space; Star, congestion; Arrow, pyknotic nuclei; Triangle, luminal cast; Bifid arrow, vacuolation, and Bold arrow, lymphatic infiltration.

Sections of the kidney from the control group revealed that the renal cortex was packed with renal (Malpighian) corpuscles, glomerular capillaries, Bowman’s capsules, and urine space. Proximal convoluted tubules have a tiny lumen surrounded by cubical cells with round nuclei at the base of the tubule. The lumen of the distal convoluted tubules was large and bordered by simple cubical cells with sphere nuclei in the middle or at the apex of the tubule walls (Figure 7A). As shown in Figure 7B, renal sections of the Vit. C group showed a normal appearance of most renal glomeruli and tubules with no lymphatic intrusion. The glomeruli are normal and standard size inside Bowman’s capsule. In Figure 7C, a microscopic examination of kidney sections from the linezolid group exhibited some vacuolated, fractured, and swollen glomeruli (expansion of glomeruli size inside Bowman’s capsules and decreasing capsular space volume). In the proximal and distal convoluted tubules, cell nuclei exhibited complete or partial damage, implying dissolution. Additionally, the nucleus has been observed to compact (pyknosis). There was mild inflammatory cell infiltration, a moderate degree of congestion, and mild hemorrhagic areas were seen. Within tubular cells, cellular remains were found (lumen cast). Figure 7D reveals that renal sections of the linezolid + Vit. C group exhibited some tubular epithelial cells in renal convoluted tubules with mild vacuolation and degenerative changes. The glomerulus is of normal size within Bowman’s capsules, with a normal volume of capsular space. Other glomeruli were fragmented, vacuolated, and swollen at a mild rate. Few pyknotic nuclei were seen in only a few tubules. There was no evidence of lymphatic invasion. There was no area of hemorrhage. Congestion was noted to be moderate. No luminal cast was seen.

In conclusion, the LZD + Vit. C group showed fewer effects than the LZD group as it showed more progress in renal tissue configuration. The severities of the morphological changes in the kidney after different treatments are shown in Table 3.

**TABLE 3:** Semi-quantitative analysis of histology of rat kidneys.

Groups	Lymphatic infiltration	Hemorrhage	Tubular degeneration	Congestion	Fragmented glomeruli	Swollen glomeruli	Vacuolated glomeruli
Control	−	−	−	−	−	−	−
Vit. C	−	−	−	−	−	−	−
Linezolid	+	+	++	++	++	++	**++**
Linezolid + Vit. C	−	−	+	++	+	+	−

(−) indicates normal, (+) indicates mild, and (++) indicates moderate.

### 3.7 CD68 immunohistochemical detection in rat kidney

**FIGURE 8:**
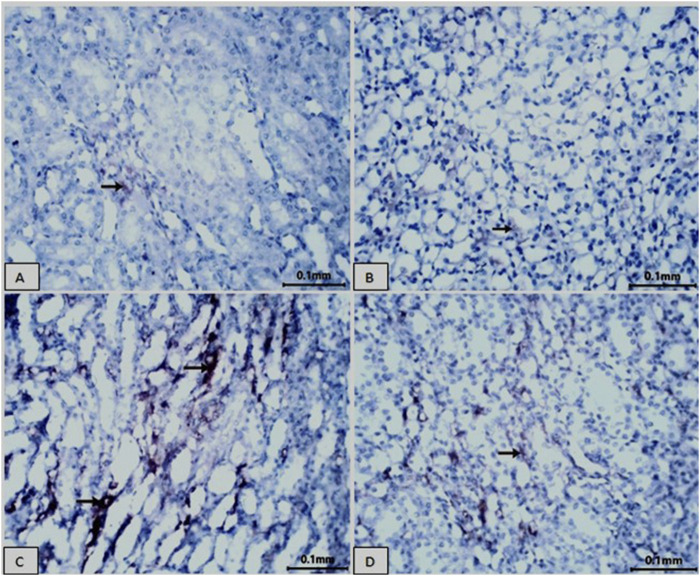
Showing immunohistochemical staining for CD68 in rat kidney tissue (scale bar = 0.1 mm). Groups **(A)** normal control and **(B)** Vit. C showed no CD68 expression. In contrast, the LZD group **(C)** displayed a significant increase in CD68 immunoreactivity within the cytoplasm of the proximal tubular cells. The co-treatment with Vit. C and LZD **(D)** resulted in an obvious reduction of CD68 immunostaining compared to the linezolid group. Brown coloration refers to positive linezolid staining.

Figure 8 and Table 4 present immunohistochemical information about Groups 1(8A) and 2(8B), the control and Vit. C-treated animals, respectively: Examination of kidneys of male rats in these groups showed rare CD68-positive macrophages in the tubular interstitium and absence of macrophage infiltration in the areas of the glomeruli; also, negative immunoreactivity for CD68 in the cytoplasm of tubular epithelial cells was found (Figure 8A). Figure 8C shows that LZD-treated animals exhibited CD68-positive cells in some renal tubules, moderate CD68-positive reaction in cells of some renal tubules, and a strong CD68-positive reaction in the tubular interstitium appeared in the kidney tissues of male rats of Group 3. Kidney sections of Group 4 (Figure 8D; LZD + Vit. C) showed a faint immunoreactivity for CD68 in the interstitial tissue and moderate CD68-positive in some tubules, reflecting the role of Vit. C treatment against the toxic effect of LZD.

**TABLE 4:** Image analysis of %area of rat kidney immunohistochemically stained by CD68.

Groups	Mean area% ± SE
Control normal	0.089 ± 0.0319
Vit. C	0.076 ± 0.0142
LZD	3.655 ± 0.314^a^
LZD + Vit. C	0.313 ± 0.0532^ab^

The CD68 +Ve immune reaction was measured within 10 non-overlapping fields/sections for each animal. Scale bar = 10 µm. (a) indicates significance compared to the normal group, (b) indicates significance compared to the LZD group.

## 4 Discussion

This research investigated the protective role of vitamin C against the hepatotoxic and nephrotoxic effects induced by linezolid in rats. The study unveiled several LZD toxicodynamics, including oxidative stress, inflammation, autophagy downregulation, and apoptosis, which resulted in tissue damage. As demonstrated by diminishing oxidative stress markers, suppressing inflammatory mediators (IL-1β and TNF-α), and modulating Wnt-related gene expression, vitamin C administration was found to alleviate such negative effects while boosting antioxidant activity and preserving liver and kidney damage integrity brought on by using linezolid. Because of its presumably unique molecular target, linezolid works effectively against bacteria that are resistant to a range of antibiotics (Abd El Latif et al., 2019).

### 4.1 Lactic acidosis

The present study revealed that linezolid induced high serum lactic acid levels. Treatment with linezolid has been associated with lactic acidosis, particularly following extended administration (≥28 days). This condition represents a significant adverse drug reaction that may lead to multiorgan failure and potentially fatal outcomes (Mao et al., 2018). Linezolid-induced lactic acidosis may result from the drug’s interference with mitochondrial ribosomes, inhibiting protein synthesis, reducing respiratory chain enzymes, and accelerating glycolysis that leads to lactate overproduction independent of tissue hypoxia (Santini et al., 2017). Our results revealed that simultaneous administration of vitamin C and linezolid resulted in the reduction of lactic acid acidosis. Vitamin C enters mitochondria in its oxidized form, dehydroascorbic acid (DHA), via glucose transporter 1 (Glut l) and shields mitochondria from oxidative damage (Kc et al., 2005). It has been reported that ascorbate-deficient mice had a reduction of hepatic mitochondrial proteins (Aumailley et al., 2022). Therefore, it is evident that oxidative stress is linked to the significant rise in serum lactic acid levels caused by LZD.

### 4.2 Oxidative stress

As linezolid inhibits mitochondrial protein synthesis, it may alter its own metabolism, resulting in a higher chance of non-negligible toxicity (Di Paolo et al., 2010). Thrombocytopenia, anemia, neuropathy, and lactic acidosis are only a few of the negative consequences that can result from unstable reactive oxygen species (ROS) molecules (Kendir-Demirkol et al., 2023). Linezolid impaired energy production and increased the generation of ROS (Heidari and Khalili, 2023).

The present study found that linezolid induced a state of oxidative stress in both liver and kidney tissues via increasing hepatic and renal NO and MDA content at the expense of tissue GSH and serum catalase activity, which was found to be depleted. Therefore, linezolid can induce oxidative stress by disrupting the balance between ROS production and antioxidant defense in hepatic or renal cells. An imbalance between antioxidants and oxidants directs to the state of oxidative stress, which has the potential to cause damage to biological molecules, including proteins, carbohydrates, lipids, and DNA (Demirci-Cekiç et al., 2022).

Antioxidants are crucial in fighting oxidative stress as they neutralize or remove free radicals through electron donation (Zhang et al., 2023). The present results showed that co-administration of vitamin C with linezolid enhances the antioxidant defense mechanism via elevating catalase activity as well as hepatic and renal GSH content. In addition, it reduced both MDA and NO production in the liver and kidney. Ascorbic acid primarily donates single hydrogen atoms, a characteristic that decreases a large number of free radicals; the radical anion monodehydroascorbate mostly reacts with those radicals (Njus et al., 2020).

### 4.3 Inflammation

Regarding the inflammatory effect of LZD, the present study found that serum IL1-β as well as TNF-α in LZD-treated rats were significantly elevated compared to non-treated control rats. An *in vitro* study on bone marrow J774A.1 macrophages indicated that linezolid-induced inflammasome activation and IL-1β secretion, a property relatively unique to linezolid among other similarly acting antibiotics (He, 2012). *In vivo* studies in different acute liver failure (ALF) animal models have shown the existence of cell death by necrosis resulted in rapid IL-1α precursor release and upregulation of IL-1β and IL-18, leading to tissue injury (Chen et al., 2007; Sakurai et al., 2008; Sultan et al., 2017) with massive upregulation of anti-inflammatory molecule IL-1Ra (Gabay et al., 1997). Such effect may happen due to a dramatic decline of hepatic inhibitor of kappa B (IκB) levels and nuclear factor kappa (NF-κB) pathway activation, leading to IL-6 and TNF-α secretion, which enhances apoptosis with subsequent liver damage and animal death (Sultan et al., 2017).

TNF-α is a key cytokine in liver inflammation and disease. It induces hepatocyte apoptosis, necroptosis, and liver regeneration, and anti-TNF therapies that target specific TNF-α receptors are efficient for improvement (Tiegs and Horst, 2022). Mitochondrial dysfunction increases ROS expression, inducing liver and kidney damage with vascular remodeling, which promotes inflammation that exacerbates liver conditions and damages tissue integrity. Inflammation also results in enhancing angiogenesis and lymph angiogenesis, similar to what happens in chronic liver disease or in drug-induced acute liver failure models (Banerjee et al., 2023). Mitochondrial dysfunction in kidneys produces oxygen radicals, increasing oxidative stress that contributes to kidney injury and complications (Gyurászová et al., 2020). The present results revealed that concomitant treatment with vitamin C and linezolid reduced serum levels of IL-1β and TNF-α to near-normal levels. Antioxidants can mitigate oxidative stress and inflammatory pathways similar to curcumin, vitamin C, and others. Understanding cytokine-antioxidant interplay aids therapeutic development (Bhol et al., 2024).

### 4.4 Autophagy

This is the first study to investigate the effect of linezolid on Beclin-1 involved in autophagy regulation. Treatment of rats with LZD resulted in the reduction of Beclin-1, while co-administration of vitamin C enhanced its level to a near-normal control level. Beclin-1 is integral to both liver physiology and pathophysiology, especially concerning the regulation of autophagy. Elevated Beclin-1 levels facilitated hepatocyte regeneration by promoting autophagy (Han et al., 2016). Furthermore, in the context of lipopolysaccharide-induced sepsis, apilarnil, a bee-derived product, demonstrated a potential protective effect by enhancing Beclin-1 expression and modulating autophagy within the liver (Doğanyiğit et al., 2020). Additionally, both energy restriction and resveratrol treatment have been shown to stimulate autophagy in hepatic steatosis via a significant augmentation of autophagy-related proteins, including Beclin-1 (Milton-Laskibar et al., 2018). Collectively, these findings indicate promising therapeutic strategies that could target these pathways to promote liver health and regeneration. In addition, endogenous Beclin-1 expression was found to be protective against acute kidney injury (AKI) by attenuating damage, promoting tubular cell proliferation, and reducing fibrosis (Li P. et al., 2020; Shi et al., 2022). Beclin1 was investigated as a key factor for vitamin C to regulate cell cycle and autophagy (Ren et al., 2021).

### 4.5 Wnt signaling

Our results revealed that LZD induced Wnt 7a and Wnt 10a gene expression compared to the control group. In contrast, co-administration of vitamin C with LZD lowered their expression. This was the first study to unveil the effect of LZD on Wnt gene expression.

Wnt signaling is particularly important in regulating cell proliferation and differentiation, cell polarity and migration, and the development of the cardiovascular, nervous, and mammary systems. As a result, it is not surprising that improper activation causes a wide range of human diseases (Herr et al., 2012). Wnt signaling activation is critical in liver regeneration, metabolic zonation, liver diseases, and cancer. Hyperactivation of Wnt signaling promotes liver tumorigenesis and the progression of liver disease (Zou and Park, 2023). Both canonical and non-canonical Wnt pathways have been reported to play a key role in the development of non-alcoholic fatty liver disease (NAFLD) that has been improved by atorvastatin via suppression of Wnt/β-catenin pathway (Sui et al., 2020). Niclosamide has been found to provide a protective effect by blocking glutaminolysis and the Wnt/β-catenin signaling pathway linked to liver fibrosis (El-Ashmawy et al., 2020; Esmail et al., 2021). These results demonstrate how therapeutic approaches for liver illnesses may involve targeting Wnt signaling.

Concerning the role of Wnt signaling dysregulation in renal impairment, it has been reported that renal fibrosis can be alleviated, and kidney function can be enhanced by blocking the Wnt/β-catenin pathway. By altering Wnt pathway genes, ipafricept, a decoy receptor for Wnt ligands, has demonstrated potential in the treatment of solid cancers (Jimeno et al., 2017). Through reducing Wnt/β-catenin expression, qingshen granules, metformin, or shenkang injection (SKI) have been shown to lessen renal fibrosis (Wang et al., 2019; Mohamed, 2021; Wei et al., 2022).

Through blocking the Wnt/β-catenin signaling pathways, vitamin C has been found to promote bone regeneration (Choi et al., 2019), suppress pancreatic tumor growth (Kim et al., 2022), and control oxidative stress in zebrafish (Liu et al., 2021). Additionally, vitamin C plays a function in determining cell fate by promoting neurogenesis in brain progenitor cells via redox-sensitive Wnt/β-catenin signaling (Rharass et al., 2017).

### 4.6 Liver and kidney functions and histopathological findings

Approximately 30% of the LZD is excreted unchanged by the kidneys, while the rest is metabolized by oxidizing its morpholine ring, which is facilitated by ROS using NADPH in the liver (Wynalda et al., 2000), resulting in the creation of two metabolites: amino-ethoxy-acetic-acid and hydroxyethyl glycine (Nukui et al., 2013).

The present study showed that linezolid-induced liver damage was demonstrated by an increase in the marker enzymes alkaline phosphatase (ALP), aspartate aminotransferase (AST), and alanine aminotransferase (ALT). Lower plasma albumin concentration and markedly elevated serum urea and creatinine levels all indicated issues with renal function. These effects were associated with histopathological findings in both hepatic and renal tissues. This harmful effect of LZD on the liver and kidney is a result of metabolic disturbances, lactic acidosis, oxidative stress, dysregulated autophagy, and disrupted Wnt signaling pathways.

It has been reported that LZD induced oxidative stress, lactic acidosis, liver damage, and renal impairment in Wistar rats infected with methicillin-resistant *Staphylococcus aureus*. A histopathological investigation identified diseases such as inflammation, vacuolization, steatosis, and hepatocyte apoptosis, which were reversed by using silymarin (Vivekanandan et al., 2018). Vitamin E and garlic restored antioxidant balance in the liver and renal dysfunctions caused by LZD (Abd El Latif et al., 2019). Concerning the effect of LZD on renal function, a previous study revealed that using 50 mg of linezolid for 7 days caused mild renal impairment, oxidative stress, and damage to tubular epithelial cells (Hindawy and Hendawy, 2019). Clinically, within 10 days of starting LZD medication, pediatric patients between the ages of 6 and 17 showed signs of renal impairment, elevated liver enzyme levels, and thrombocytopenia (Bayram et al., 2017). Therefore, it was strongly advised to regularly monitor platelet counts, liver enzyme levels, and kidney function during linezolid therapy, especially in children and individuals with pre-existing renal issues (Bayram et al., 2017; Kawasuji et al., 2021).

According to the current study, the histological findings indicate severe hepatic injury characterized by lobular disorganization, apoptosis, steatosis, and disruption of normal hepatic architecture. Additional features include venous congestion and lymphocytic proliferation, pointing to chronic inflammation. The histopathological examination of kidneys belonging to LZD-treated rats revealed significant damage, edema, inflammation, tubular damage, mild inflammatory infiltration, moderate congestion, and mild hemorrhages. These findings suggest that LZD induced nephrotoxicity due to ischemia and inflammation.

Along with being an effective antioxidant that eliminates ROS and a free-radical scavenger, vitamin C is a reducing agent involved in tissue integrity, collagen manufacture, neuroprotection, hematopoiesis, and leukocyte function. In association, histological examination of the liver revealed substantial recovery, with restored hepatocyte health, reduced congestion, absence of inflammation, and resolution of fatty degeneration compared to the LZD-treated group. In addition, the histology of the kidneys from the LZD + Vit. C group showed mostly normal glomeruli associated with mild swelling and fragmentation. Tubules were found to be limited to mild vacuolation and few pyknotic nuclei without lumen casts. Vascular changes include moderate congestion but no hemorrhage or inflammatory infiltration. Overall, the current findings reflect the reduced severity of renal pathology as compared to the LZD-treated group.

Historically, CD68 has been utilized as an important cytochemical marker for the immunostaining of monocytes and macrophages in the histochemical examination of inflamed tissues, tumor specimens, and other immunohistopathological contexts (Chistiakov et al., 2017). Immunohistochemical detection of CD68 in kidney sections revealed that the linezolid-treated group exhibited a notable enhancement in CD68 immunoreactivity localized in the cytoplasm of proximal tubular cells. In contrast, the combination treatment of vitamin C and linezolid led to a significant decrease in CD68 immunostaining when compared to the linezolid-treated group. CD68 is a glycoprotein predominantly found in macrophages and various mononuclear phagocytes. CD68 serves as a marker for macrophages and plays a significant role in the development of numerous renal disorders, especially when pro-inflammatory cytokines such as TNF-α and IL-1β are present. This interaction may intensify kidney damage and promote fibrosis. Elevated levels of CD68 expression have been reported to be associated with lupus nephritis and diabetic nephropathy. Higher CD68 levels in renal interstitium are associated with worse renal outcomes, indicating a link between macrophage activity and disease progression (Dias et al., 2017).

Targeting the pathways involving CD68, TNF-α, and IL-1β may offer therapeutic avenues to mitigate renal inflammation and fibrosis. The balance between these cytokines and their inhibitors is crucial for maintaining renal health (Descamps-Latscha et al., 1995)

## 5 Conclusion

This research underscores the protective role of vitamin C in mitigating oxidative stress, inflammation, and tissue damage induced by linezolid in liver and kidney tissues. The administration of linezolid was found to impair mitochondrial function, intensify oxidative stress, and activate inflammatory pathways, resulting in notable biochemical and histopathological alterations. Vitamin C was effective in alleviating these detrimental effects by bolstering antioxidant defenses, decreasing levels of inflammatory markers such as IL-1β and TNF-α, and restoring the integrity of the tissues. Furthermore, the concurrent use of vitamin C was shown to modulate autophagy-related proteins (Beclin-1), inhibit the dysregulation of Wnt signaling, and normalize CD68 expression, highlighting its therapeutic efficacy. These results support the incorporation of antioxidants like vitamin C in strategies aimed at mitigating linezolid-induced toxicity, providing valuable insights for potential clinical applications.

## Data Availability

The datasets presented in this study can be found in online repositories. The names of the repository/repositories and accession number(s) can be found in the article/supplementary material.
